# Maternal and Fetal Outcomes Associated With Scrub Typhus in Pregnancy: A Systematic Review

**DOI:** 10.7759/cureus.114195

**Published:** 2026-08-09

**Authors:** Vaishnavi Gawande, Sakshi Sharma, Tithi Shah, Divyansh Sharma

**Affiliations:** 1 Community and Family Medicine, All India Institute of Medical Sciences, Bhopal, Bhopal, IND; 2 Community Medicine, Armed Forces Medical College, Pune, IND

**Keywords:** fetal outcome, maternal outcome, orientia tsutsugamushi, pregnancy-related outcomes, scrub typhus, systematic review

## Abstract

Scrub typhus, caused by *Orientia tsutsugamushi* and transmitted by larval trombiculid mites, is endemic across much of the Asia-Pacific region. It is frequently missed or misdiagnosed in pregnant individuals, where its consequences may be severe. Despite the severe maternal and fetal risks associated with scrub typhus, a comprehensive synthesis of the infection’s total burden during pregnancy remains a critical gap in the literature. We searched five bibliographic databases (PubMed, CINAHL, MEDLINE Ultimate, ClinicalKey, and Ovid Embase), four trial registries (World Health Organization International Clinical Trials Registry Platform, ClinicalTrials.gov, the EU Clinical Trials Register, and the ISRCTN Registry), and two additional sources (Google Scholar, searched as a source of grey literature, and the One Nation One Subscription (ONOS)) platform for English-language studies published between January 1, 2015 and December 31, 2025, that reported prevalence or maternal or fetal outcomes among pregnant women with laboratory-confirmed scrub typhus. Two reviewers independently screened records, extracted data, and appraised quality using the design-appropriate Joanna Briggs Institute checklists. Eligible studies were few, small, and clinically heterogeneous; the findings were synthesized narratively in accordance with the Synthesis Without Meta-analysis guidance and reported in accordance with the Preferred Reporting Items for Systematic Reviews and Meta-Analyses 2020. In total, 23 studies were eligible, reporting on 434 pregnant women with confirmed scrub typhus. A total of 18 studies were from India. Overall, 12 maternal deaths were recorded across seven studies; the largest cohort (n = 260, China) reported none. Intensive care admission ranged from 3.1% to 69.7%, and multiorgan dysfunction, hepatic and renal injury, and respiratory failure were the major maternal complications. Total fetal loss ranged from 0% to 42.4% and preterm birth from 0% to 44.4%. Earlier gestational age at infection and longer delay before treatment both predicted worse outcomes. Azithromycin was the mainstay of treatment and, in the largest cohort, was associated with improved fetal survival. All available evidence was from facility-based studies, suggesting that outcomes are driven primarily by early clinical recognition and timely initiation of appropriate treatment, and that the true community-level burden remains unknown. In endemic settings, empirical azithromycin may be considered when scrub typhus is clinically suspected, and laboratory confirmation is likely to be delayed, although this observation is based on limited, single-center data and warrants confirmation in prospective studies.

## Introduction and background

Scrub typhus is caused by *Orientia tsutsugamushi*, an obligate intracellular bacterium transmitted to humans through the bite of infected larval trombiculid mites, or chiggers. It is endemic to the “tsutsugamushi triangle,” a region from Afghanistan and Pakistan in the west, across South and Southeast Asia and southern China, up to the Russian Far East, and down into northern Australia. Approximately a billion people live at risk within this zone, and a million are infected each year, making scrub typhus one of the most common yet neglected causes of acute undifferentiated fever in the Asia-Pacific region [1,2].

The clinical presentation of scrub typhus is variable, ranging from mild, self-limiting fever to rapid progression toward multiorgan failure. The one specific sign, an eschar at the site of mite bite, is found in only a minority of cases, and the early clinical profile of fever, headache, and myalgia is indistinguishable from malaria, dengue, enteric fever, and leptospirosis, all of which are endemic in the same region [3,4]. Untreated, the disease has a high mortality rate; treated promptly with an effective antimicrobial, it reduces mortality [5].

Pregnancy further complicates its clinical course. The immunological adaptations required to sustain the semi-allogeneic fetus, a shift toward T-helper-2 dominance and an attenuation of cell-mediated immunity, may impair the containment of intracellular organisms such as *O. tsutsugamushi*. At the same time, the physiological hypercoagulability of pregnancy increases the risk of severe systemic infection escalating into disseminated intravascular coagulation (DIC). The reduced colloid osmotic pressure of pregnancy lowers the threshold for pulmonary edema [6]. These severe maternal complications frequently compromise fetal outcomes, leading to high rates of miscarriage, stillbirth, preterm delivery, and fetal growth restriction [4,7].

Despite these severe risks, evidence on scrub typhus in pregnancy remains scattered across single-case reports and small hospital studies, which use different diagnostic tests and report outcomes inconsistently [4,7]. Given the fragmented and heterogeneous nature of existing research, a comprehensive synthesis is needed to understand the burden and outcomes of scrub typhus in pregnancy. To address this gap, we conducted a systematic review of scrub typhus infection during pregnancy to characterize adverse effects among this population.

The primary objective was to synthesize the maternal and fetal outcomes reported in association with laboratory-confirmed scrub typhus in pregnancy. Secondary objectives were to describe the prevalence of infection among febrile pregnant women in studies, to summarize the diagnostic methods and treatment regimens used, and to identify factors reported to predict poor maternal or fetal outcomes.

## Review

Methodology

Protocol and Registration

The protocol was registered on the Open Science Framework (DOI 10.17605/OSF.IO/GCXR5), and the review is reported in accordance with the Preferred Reporting Items for Systematic Reviews and Meta-Analyses (PRISMA) 2020 guidelines [8] (Supplementary Material S5) and the Synthesis Without Meta-analysis (SWiM) reporting guideline [9] (Supplementary Material S6). Study quality was appraised using the Joanna Briggs Institute (JBI) critical appraisal tools.

Eligibility Criteria

Eligibility was defined using a PECO-S framework (Population, Exposure, Comparator, Outcomes, Study design).

Population: Pregnant women of any age with laboratory-confirmed scrub typhus. Diagnosis of scrub typhus was considered confirmed when supported by one or more of the following laboratory tests: IgM enzyme-linked immunosorbent assay (ELISA), indirect immunofluorescence assay (IFA), the Weil-Felix test, immunochromatographic or rapid diagnostic test, polymerase chain reaction (PCR), including real-time or nested PCR or next-generation sequencing.

Exposure: Scrub typhus infection during pregnancy.

Comparator: None; both comparative and non-comparative designs were eligible.

Outcomes. Studies that reported at least one of the following: the prevalence of scrub typhus among pregnant women, maternal outcome, or fetal outcome.

Study designs. All observational designs were eligible: retrospective and prospective cohort studies, cross-sectional studies, case series, and case reports.

Exclusion criteria: We excluded studies without laboratory confirmation; those in non-pregnant populations or in which a pregnant subgroup could not be separated; studies reporting no maternal or fetal outcome; narrative, scoping, and systematic reviews; pooled analyses, editorials, commentaries, letters, and conference abstracts; experimental studies; and non-English articles.

Information Sources and Search Strategy

A literature search was conducted across five bibliographic databases: PubMed, CINAHL (via EBSCOhost), MEDLINE Ultimate, ClinicalKey, and Ovid Embase; and four trial registries: the World Health Organization (WHO) International Clinical Trials Registry Platform (ICTRP), ClinicalTrials.gov, the EU Clinical Trials Register, and the ISRCTN Registry. Two additional sources were searched: Google Scholar, as a source of grey literature, and the One Nation One Subscription (ONOS) portal.

The search strategy combined controlled vocabulary (e.g., MeSH, Emtree, and CINAHL Subject Headings, where available) with free-text terms related to scrub typhus (*O. tsutsugamushi*), pregnancy, and maternal and fetal outcomes. Search strategies were tailored to the indexing system and search syntax of each information source. Searches were limited to English-language publications from January 1, 2015, to December 31, 2025. As Google Scholar and the ONOS portal do not support Boolean or field-tag searching, all records retrieved from these two sources were screened manually in full against the same eligibility criteria applied to records from the databases and registries, rather than being limited to a relevance-ranked subset. The complete database-specific search strategies are provided in Supplementary Material S1. The reference lists of included studies were manually screened to identify any further eligible records.

Study Selection

All retrieved records were imported into a reference manager and de-duplicated. Screening was conducted in Rayyan with blinding enabled. Two reviewers (TS and DS) independently screened titles and abstracts against the predefined eligibility criteria. Disagreements were resolved through discussion with a third reviewer (VG). Full texts of the remaining records were retrieved and independently assessed against the eligibility criteria by two reviewers (DS and TS), and discrepancies were resolved by two reviewers (VG and SS).

Data Extraction

Data were independently extracted by two reviewers using a piloted Microsoft Excel data extraction sheet. Extracted variables included study characteristics (author, year, country, and setting), study design, sample size, diagnostic method, maternal age, gestational age and trimester at diagnosis, treatment received, prevalence, and all predefined maternal and fetal outcomes. Discrepancies were resolved by discussion and consensus. The complete extraction dataset is provided in Supplementary Material S3.

Outcomes that were explicitly reported and quantifiable within the original studies were extracted. Clinical findings mentioned descriptively but not identified or enumerated as predefined outcomes (e.g., elevated liver enzymes not classified as hepatitis or respiratory symptoms not reported as acute respiratory distress syndrome (ARDS) were recorded as not reported rather than inferred. Where fetal loss was reported only as a combined outcome (e.g., miscarriage and intrauterine fetal death) without separate estimates, the composite measure was retained as reported, and this limitation was documented. For studies in which scrub typhus constituted a subgroup of a broader cohort (e.g., febrile illness or maternal mortality cohorts), only data specifically attributable to scrub typhus were extracted; where attribution was not possible, this was explicitly noted.

Outcome Definitions

The predefined maternal outcomes included maternal death, intensive care unit (ICU) admission, ARDS or documented respiratory distress, hepatitis or hepatic dysfunction, multiorgan dysfunction syndrome (MODS), acute kidney injury (AKI), and coagulopathy or DIC. Predefined fetal and neonatal outcomes comprised total fetal loss, intrauterine fetal death or stillbirth, spontaneous abortion, preterm birth (<37 completed weeks of gestation), low birth weight (<2,500 g), neonatal death, and neonatal intensive care unit (NICU) admission. Where available, predictors of adverse maternal or fetal outcomes and the prevalence of scrub typhus among pregnant women were also extracted. Operational definitions for all outcomes are provided in Supplementary Material S4.

Risk-of-Bias (Quality) Assessment

The methodological quality of the included studies was independently assessed by two reviewers (VG and TS) using the JBI critical appraisal checklist appropriate to each study design, including checklists for cohort studies (11 items), case series (10 items), and case reports (8 items). Each item was rated as Yes, No, or Unclear. Overall study quality was categorized using predefined thresholds based on the number of JBI checklist items rated No or Unclear: low risk (0-1), moderate risk (2-3), and high risk (≥4). Disagreements were resolved through discussion, with a third reviewer (SS). Detailed quality appraisal results are presented in Supplementary Material S2.

Data Synthesis

A structured narrative synthesis was undertaken in accordance with the SWiM reporting guideline [9]. Studies were first grouped by study design and subsequently synthesized by outcome domain. Study characteristics, diagnostic methods, treatment approaches, and reported maternal and fetal outcomes were summarized in evidence tables to facilitate comparison across studies.

For each outcome, findings were synthesized descriptively using study-level counts, proportions, and ranges, without attempting to calculate pooled estimates due to substantial clinical and methodological heterogeneity. The direction, consistency, and magnitude of findings were examined across studies, while variations in reported outcomes were explored qualitatively with reference to differences in study design, study setting, sample size, diagnostic methods, treatment regimens, and timing of diagnosis and treatment initiation.

Results

Study Selection

The literature search identified 1,765 records: 1,023 from the five bibliographic databases (ClinicalKey, n = 627; MEDLINE Ultimate, n = 217; CINAHL, n = 78; PubMed, n = 70; Ovid Embase, n = 31), three from the trial registries (all from the ISRCTN registry; the ICTRP, ClinicalTrials.gov, and the EU Clinical Trials Register returned none), and 739 from the two additional sources (Google Scholar, n = 719; ONOS, n = 20). After removal of 361 duplicate records, 1,404 unique records underwent title and abstract screening, of which 1,326 were excluded. A total of 78 articles were retrieved for full-text assessment.

**Figure 1:**
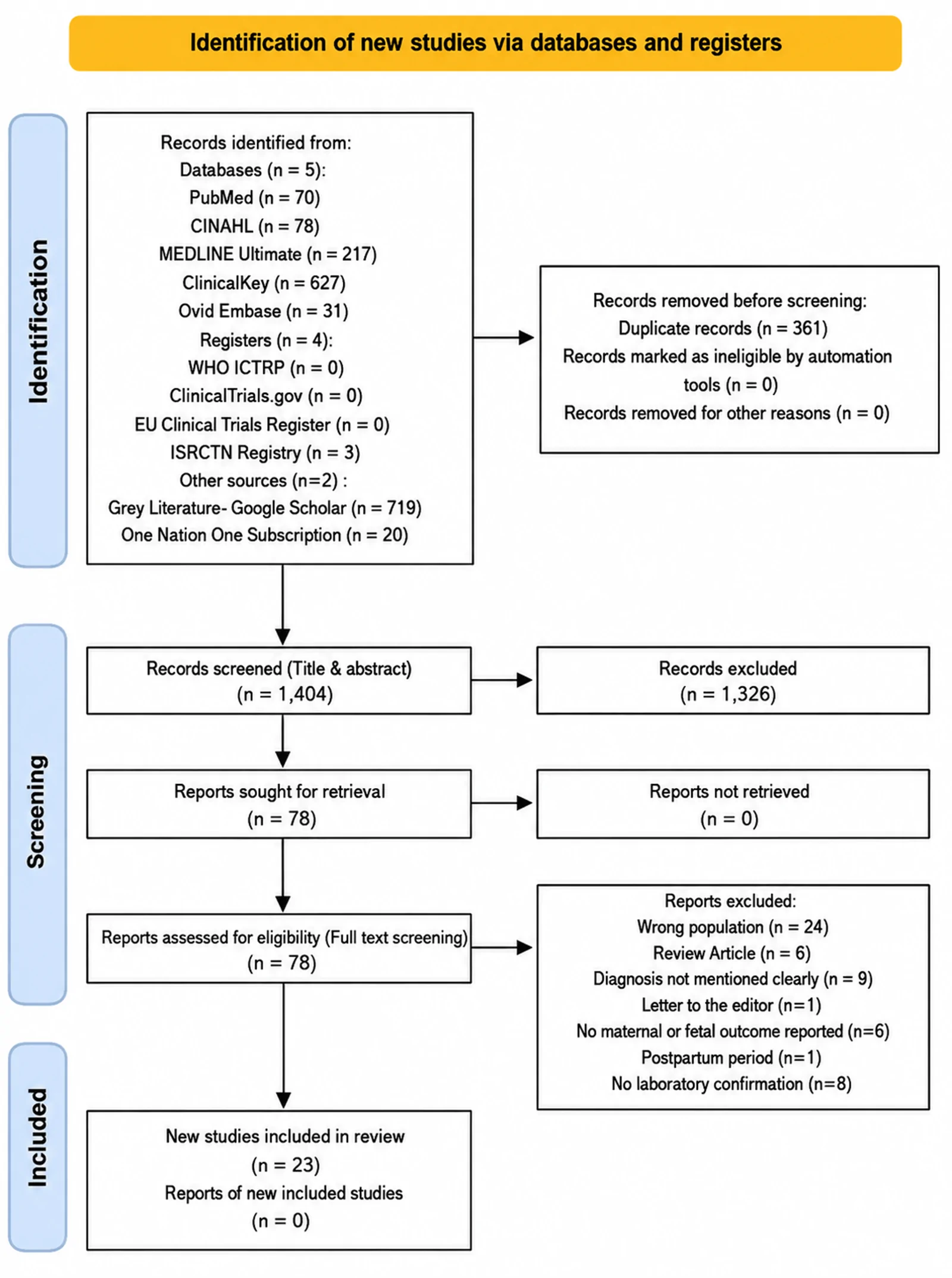
Preferred Reporting Items for Systematic Reviews and Meta-Analyses 2020 flow diagram of study identification, screening, and inclusion.

Following full-text review, 55 articles were excluded for the following reasons: non-pregnant population or inseparable pregnancy subgroup (n = 24), unclear diagnosis (n = 9), lack of laboratory confirmation (n = 8), review articles (n = 6), absence of reported maternal or fetal outcomes (n = 6), postpartum-only population (n = 1), and letter to the editor (n = 1). Consequently, 23 studies met the eligibility criteria and were included in the review [3,4,6,7,10-28]. The study selection process is presented in the PRISMA 2020 flow diagram (Figure 1).

Characteristics of Included Studies

The review included 23 studies published between 2015 and 2025, comprising data from 434 pregnant women with laboratory-confirmed scrub typhus [3,4,6,7,10-28]. The included studies comprised nine observational studies [3,4,7,10-15], five case series [16-20], and nine case reports [6,21-28]. Observational studies included retrospective cohorts, a prospective hospital-based cohort, a multicenter cohort, and a retrospective maternal mortality audit.

Most studies were conducted in India (18/23), with study sites distributed across Vellore, Chandigarh, Himachal Pradesh, Telangana, Northeast India, Uttarakhand, Uttar Pradesh, Puducherry, Chhattisgarh, and Odisha. As four of the Indian studies were conducted in Chandigarh and two in Vellore, these studies were cross-checked for overlapping enrolment periods and patient-level case characteristics to exclude the possibility of duplicate patients being counted across studies; no overlap in study duration or case characteristics was identified, and all six studies were retained as independent cohorts. The remaining studies originated from China (Yunnan and Guangxi provinces) [7,27], Laos (Vientiane) [11], Thailand (Thailand-Myanmar border) [21], and Bhutan [25]. All included studies were conducted in healthcare facilities, predominantly tertiary or referral hospitals.

Study sample sizes ranged from single-patient case reports to a multicenter cohort of 260 pregnant women in China, the largest included study [7]. Other comparatively large cohorts included those by Rajasab and Zaki (n = 34) [15], Rajan et al. (n = 33) [4], and Yadav et al. (n = 27) [12]. Detailed characteristics of the included studies are presented in Table 1.

**Table 1:** Characteristics of included studies (N = 23). *: Halder et al.: 6 scrub typhus deaths within a 212-death all-cause audit. Composite = intrauterine death + abortion, not separable. AFI = acute febrile illness; AKI = acute kidney injury; ARDS = acute respiratory distress syndrome; DIC = disseminated intravascular coagulation; ECMO = extracorporeal membrane oxygenation; ELISA = enzyme-linked immunosorbent assay; FGR = fetal growth restriction; GA = gestational age; HEV = hepatitis E virus; HP = Himachal Pradesh; ICT = immunochromatographic test; ICU = intensive care unit; IFA = immunofluorescence assay; IUD = intrauterine death; LBW = low birth weight; MODS = multiorgan dysfunction syndrome; NE = Northeast; NGS = next-generation sequencing; NICU = neonatal intensive care unit; PCR = polymerase chain reaction; PP = postpartum; RDT = rapid diagnostic test; ST = scrub typhus; T1/T2/T3 = first/second/third trimester; UP = Uttar Pradesh; wk = weeks

Study	Design	Country	n	Diagnostic method	Population (age, GA, trimester)	Key outcomes
Observational studies (n = 9)						
Halder et al. (2015) [10]	Retrospective maternal-death audit	India (Vellore)	6*	Cause of death classification	Mortality audit; ST = 6/212 maternal deaths	6 maternal deaths
Chansamouth et al. (2016) [11]	Prospective cohort	Laos	9 (of 250)	RDT, IgM IFA, PCR, culture	Febrile inpatients ≥37.5°C; ST 3.6%	No deaths, 2 fetal losses (22%), 1 preterm, 1 LBW
Kumar et al. (2016) [3]	Prospective cohort	India (HP)	14	IgM ELISA	Rural; GA 15–34 wk; T2 = 8, T3 = 6	No deaths, no fetal loss; all term. ARDS 4, MODS 4. Early azithromycin treatment
Rajan et al. (2016) [4]	Retrospective cohort	India (South)	33	Clinical + IgM ELISA	T1 = 5, T2 = 9, T3 = 19; prevalence 4.5%	1 death, ICU 23 (69.7%), fetal loss 14 (42.4%, composite), preterm 3
Yadav et al. (2023) [12]	Retrospective cohort	India (Chandigarh)	27	IgM ELISA	GA 10–38 wk; T1 = 1, T2 = 2, T3 = 20, PP = 4; prevalence 7.1%.	1 death, ICU 8 (29.6%), fetal loss 5, preterm 12 (44.4%), LBW 8/19
Jamil et al. (2024) [13]	Retrospective study	India (NE)	9	Weil–Felix/ICT	T1 = 2, T2 = 2, T3 = 5; no eschar	No deaths, MODS 2, fetal loss 1, preterm 1
Thomas et al. (2024) [14]	Prospective AFI cohort	India (Vellore)	9 (of 180)	IgM ELISA ± eschar	ST subgroup of the AFI cohort	No deaths, ICU 2, fetal loss 1
Rajasab and Zaki (2025) [15]	Retrospective cohort	India (Telangana)	34	WHO criteria + IgM ELISA	GA 10–38 wk; T1 = 6, T2 = 9, T3 = 19; prevalence 12.3%	No deaths, ICU 15 (44.1%), fetal loss 12 (35.3%, composite), preterm 6
Zhao et al. (2025) [7]	Retrospective multicentre cohort	China (Yunnan)	260	Weil–Felix/IFA/PCR/isolation	Median GA 24 wk; T1 = 39, T2 = 121, T3 = 100	No deaths, ICU 8 (3.1%), fetal loss 57 (21.9%), preterm 33. Macrolides 82.3%
Case series (n = 5)						
Meena et al. (2016) [16]	Case series	India (Chandigarh)	6	IgM ELISA	Atypical; GA 17–41 wk	1 death, hepatitis 4, fetal loss 3, preterm 2, LBW 2
Sheokand et al. (2021) [17]	Case series	India (Chandigarh)	3	ICT/Serology	Monsoon season: mild to severe	No deaths, ICU 1, fetal loss 1, preterm 1, LBW 1
Arora (2024) [18]	Retrospective case series	India (UP)	8	Serum IgM ELISA	GA 19–39 wk; 5 of 8 T3; eschar 3 of 8	No deaths, ICU 3, fetal loss 2, preterm 1
Bahadur et al. (2024) [19]	Prospective case series	India (Uttarakhand)	5	RDT	GA 27–38 wk; 4 of 5 T3; unbooked	1 death, ICU 1, fetal loss 1, preterm 2; NICU 2
Selvan et al. (2024) [20]	Case series	India (Odisha)	2	IgM ELISA	Both FGR, T2, and T3	No deaths, no fetal loss, preterm 2, LBW 2, NICU 2
Case reports (n = 9)						
Watthanaworawit et al. (2016) [21]	Case report	Thailand	1	RDT + serology	31 wk; agricultural worker	Survived; ICU, ARDS, MODS, DIC, stillbirth
Verma et al. (2017) [6]	Case report	India	1	IgM ELISA + PCR	31 wk; HEV co-infection	Died: hepatitis, MODS, DIC, stillbirth
Gupta et al. (2021) [22]	Case report	India (UP)	1	IgM ELISA + eschar	32 wk; fetal demise at diagnosis	Survived; ICU, ARDS, MODS, AKI, IUD
Rengaraj et al. (2021) [23]	Case report	India (Puducherry)	1	RT-PCR	~27 wk; postpartum diagnosis	Survived: hepatitis, AKI, neonatal death, LBW
Singh et al. (2021) [24]	Case report	India (Chhattisgarh)	1	IgM ELISA	33 wk; primigravida	Survived; ARDS, MODS, AKI, preterm, LBW
Tshering et al. (2021) [25]	Case report	Bhutan	1	IgM serology	34 wk; farmer	Survived; ARDS, hearing loss, preterm, LBW
Singla et al. (2023) [26]	Case report	India (Chandigarh)	1	ELISA	36 wk; eschar missed initially	Survived (near-miss); ICU, ARDS, MODS; stillbirth
Qin et al. (2024) [27]	Case report	China (Guangxi)	1	NGS	19 wk	Survived; ICU, ARDS, MODS, ECMO, abortion
Nazreen et al. (2025) [28]	Case report	India (Puducherry)	1	PCR	38 wk; diagnosed post-caesarean	Died; ICU, ARDS, MODS, AKI, healthy term infant

Prevalence of Scrub Typhus Among Pregnant Women

Four cohort studies reported the prevalence of laboratory-confirmed scrub typhus among febrile pregnant women presenting to healthcare facilities, with estimates ranging from 3.6% to 12.3% [4,11,12,15]. The lowest prevalence was reported from Laos (3.6%) [11], while the highest was observed in Telangana, India (12.3%; 48/391) [15]. Intermediate estimates included 4.5% (33/738) from South India [4] and 7.1% from North India [12].

Gestational Timing and Clinical Presentation

Among studies reporting gestational age at diagnosis, scrub typhus was most frequently diagnosed during the third trimester. Third-trimester cases predominated in the cohorts reported by Rajan et al. (19/33) [4], Yadav et al. (20/27) [12], Rajasab and Zaki (19/34) [15], and Jamil et al. (5/9) [13], whereas first-trimester infections were relatively uncommon. In contrast, the largest multicenter cohort reported a predominance of second-trimester infections (121/260), followed by third-trimester (100/260) and first-trimester (39/260) diagnoses [7]. The distribution of cases by trimester is summarized in Table 2.

**Table 2:** Distribution of gestational age at diagnosis among pregnant women with scrub typhus. Total reflects the six studies with complete first-, second-, and third-trimester breakdowns (Kumar et al., Rajan et al., Yadav et al., Jamil et al., Rajasab et al., Zhao et al.; 373 women). Arora, Bahadur et al., and the pooled case reports rows above are not included in this total, as they report only partial trimester data (e.g., 5 of 8, 4 of 5). GA = gestational age; wk = weeks; NR = not reported; — = not reported as a discrete count

Study	GA range	1st trimester	2nd trimester	3rd trimester	
Kumar et al. (2016) [3]	15–34 wk	0	8	6	No first-trimester cases
Rajan et al. (2016) [4]	NR	5	9	19	Third-trimester predominance
Yadav et al. (2023) [12]	10–38 wk	1	2	20	Plus 4 postpartum cases
Jamil et al. (2024) [13]	NR	2	2	5	Third-trimester predominance
Rajasab and Zaki (2025) [15]	10–38 wk	6	9	19	Third-trimester predominance
Zhao et al. (2025) [7]	Median 24 wk	39	121	100	Second-trimester predominance (largest cohort)
Arora (2024) [18]	19–39 wk	—	—	5 of 8	Majority third trimester
Bahadur et al. (2024) [19]	27–38 wk	—	—	4 of 5	Majority third trimester
Case reports [6,21-28]	19–38 wk	0	1	8	Predominantly third trimester
Total (6 studies with complete trimester data)	-	53	151	169	Second/Third trimester predominates overall

Fever was the predominant presenting symptom across the included studies and was commonly accompanied by myalgia, headache, vomiting, and abdominal pain [18]. Respiratory symptoms were also frequently reported and occasionally contributed to initial diagnostic uncertainty. Reported alternative diagnoses included pneumonia [25], coronavirus disease 2019 (COVID-19) [22], acute pyelonephritis [24], HELLP syndrome or other pregnancy-related liver disorders [23], and puerperal sepsis [17].

The presence of an eschar was variable across studies. It was absent in some case series [13,19], identified in only a minority of patients in others (3/8 [18] and 7/34 [15]), and, in two case reports, recognized only after the initial clinical assessment, resulting in delayed diagnosis and treatment [22,26].

Laboratory abnormalities were reported more consistently than clinical signs. Thrombocytopenia was a common finding, occurring in approximately one-quarter of women in the largest cohort [7] and in 29.6% of participants in the study by Yadav et al. [12], with platelet counts as low as 14 × 10⁹/L reported in severe cases [21]. Anemia was also frequently described [3], and abnormal liver function tests were common, although these were not consistently classified or reported as hepatitis across studies.

Maternal Outcomes

Maternal outcomes were reported inconsistently across the included studies, with variation in both reporting frequency and outcome definitions. In three studies, complications attributable specifically to scrub typhus could not be distinguished from those reported within broader cohorts of febrile illness or maternal mortality [10,11,14]. Study-level maternal outcomes are summarized in Table 3.

**Table 3:** Maternal outcomes reported across included studies. AKI = acute kidney injury; ARDS = acute respiratory distress syndrome; CRRT = continuous renal replacement therapy; DIC = disseminated intravascular coagulation; ECMO = extracorporeal membrane oxygenation; HEV = hepatitis E virus; ICU = intensive care unit; LFT = liver function test; MODS = multiorgan dysfunction syndrome; NR = not reported

Study	n	Death	ICU	ARDS	MODS	AKI	DIC	others
Halder et al. (2015) [10]	6	6	NR	NR	NR	NR	NR	Audit-level only
Chansamouth et al. (2016) [11]	9	0	NR	NR	NR	NR	NR	Not disaggregated for ST
Kumar et al. (2016) [3]	14	0	NR	4	4	5	NR	Hepatic dysfunction 11; sepsis 10
Rajan et al. (2016) [4]	33	1	23	NR	5	NR	NR	Hepatosplenomegaly 4
Yadav et al. (2023) [12]	27	1	8	1	4	2	NR	Hyperbilirubinemia 13
Jamil et al. (2024) [13]	9	0	NR	NR	2	NR	NR	Hepatitis 1; eclampsia 1
Thomas et al. (2024) [14]	9	0	2	NR	NR	NR	NR	Not broken out for ST
Rajasab and Zaki (2025) [15]	34	0	15	2	NR	3	NR	Jaundice 5; pneumonia 4
Zhao et al. (2025) [7]	260	0	8	NR	NR	35	2	Hepatic dysfunction 105; pneumonia 33
Meena et al. (2016) [16]	6	1	≥2	2	2	2	1	Hepatitis 4
Sheokand et al. (2021) [17]	3	0	1	1	1	1	1	Deranged LFTs
Arora (2024) [18]	8	0	3	2	1	2	NR	Not separately reported
Bahadur et al. (2024) [19]	5	1	1	1	1	0	NR	Deranged LFTs in all 5
Selvan et al. (2024) [20]	2	0	0	1	1	1	NR	Hepatitis 1
Watthanaworawit et al. (2016) [21]	1	0	1	1	1	NR	1	Septic shock
Verma et al. (2017) [6]	1	1	1	NR	1	NR	1	Acute liver failure (HEV)
Gupta et al. (2021) [22]	1	0	1	1	1	1	0	Hypoxemic respiratory failure
Rengaraj et al. (2021) [23]	1	0	NR	0	NR	1	NR	Hepatitis; HELLP mimic
Singh et al. (2021) [24]	1	0	NR	1	1	1	NR	Pneumonia; hepatomegaly
Tshering et al. (2021) [25]	1	0	NR	1	NR	NR	NR	Permanent hearing loss
Singla et al. (2023) [26]	1	0	1	1	1	NR	NR	Near-miss; thrombocytopenia
Qin et al. (2024) [27]	1	0	1	1	1	NR	NR	ECMO + CRRT
Nazreen et al. (2025) [28]	1	1	1	1	1	1	NR	Ogilvie syndrome

Maternal death: Maternal death was reported in seven studies. Cohort and case series data documented one maternal death each in the studies by Rajan et al. (1/33) [4], Yadav et al. (1/27) [12], Meena et al. (1/6) [16], and Bahadur et al. (1/5) [19]. Two individual case reports also described fatal outcomes, one associated with acute liver failure in the setting of hepatitis E virus co-infection [6] and the other with Ogilvie syndrome and concurrent dengue infection [28]. In addition, a retrospective maternal mortality audit identified six scrub typhus-related maternal deaths among 212 maternal deaths over 10 years (2.8%) [10]. Overall, 12 maternal deaths were reported across seven studies, all conducted in India. The six deaths identified by Halder et al. were ascertained through retrospective audit of all-cause maternal mortality rather than from a defined cohort of scrub typhus pregnancies with complete outcome data; all other maternal and fetal outcomes for this study are not reported (Table 3) and are therefore not directly comparable to, or with, the denominators and event counts reported across the remaining 22 studies. In contrast, no maternal deaths were reported in the largest multicenter cohort of 260 women [7] or in several other cohort studies and case series [3,13,15].

Critical illness and organ dysfunction: The proportion of women requiring intensive care varied substantially across studies, ranging from 3.1% (8/260) in the largest multicenter cohort [7] to 69.7% (23/33) in a South Indian referral center [4]. Intermediate ICU admission rates of 29.6%, 37.5%, and 44.1% were reported by Yadav et al. [12], Arora [18], and Rajasab and Zaki [15], respectively.

MODS was reported in two cohort studies, affecting 15.2% of women in the study by Rajan et al. [4] and 14.8% in the study by Yadav et al. [12], and was also described in severe individual case reports [26,27]. Hepatic involvement was among the most frequently reported complications, occurring in 105 of 260 women in the largest cohort [7] and in 11 of 14 women in a sub-Himalayan cohort [3]. However, terminology varied and was not consistently reported as hepatitis. AKI was documented in 35 of 260 women in the largest cohort [7] and was reported less frequently in other studies [3,12,15]. ARDS was primarily described in case reports and small case series [16,17,21,27], whereas DIC was explicitly reported in only a few studies [6,7,16,17,21].

One prospective cohort of 14 pregnant women reported uniformly favorable maternal outcomes following azithromycin treatment initiated after diagnosis. No maternal deaths, fetal losses, or preterm births were observed, and all pregnancies resulted in term deliveries [3].

Fetal and Neonatal Outcomes

Fetal loss was the most consistently reported fetal outcome across the included studies, although its frequency varied considerably. Reported rates ranged from no fetal losses in a prospective cohort of 14 women [3] to 42.4% (14/33) in a South Indian cohort [4]. Intermediate estimates included 18.5% in the study by Yadav et al. [12], 21.9% (57/260) in the largest multicenter cohort [7], and 35.3% (12/34) in the study by Rajasab and Zaki [15]. Two cohort studies reported fetal loss as a composite outcome of miscarriage and intrauterine fetal death without separate estimates [4,15]. In the largest cohort, 57 fetal losses comprised 21 intrauterine fetal deaths and 36 miscarriages, with poorer fetal outcomes reported among women infected earlier in pregnancy [7]. Study-level fetal and neonatal outcomes are summarized in Table 4.

**Table 4:** Fetal outcomes reported across included studies. ^†^: Rajan et al. and Rajasab et al. reported fetal loss only as a non-separable composite of intrauterine death and abortion (—). 8/19 LB = events per live-birth denominator. IUD = intrauterine death; LB = live births; LBW = low birth weight (<2,500 g); NICU = neonatal intensive care unit; NR = not separately reported; SB = stillbirth

Study	n	Total fetal loss	IUD/SB	Abortion	Preterm	LBW	Neonatal death	NICU
Halder et al. (2015) [10]	6	NR	NR	NR	NR	NR	NR	NR
Chansamouth et al. (2016) [11]	9	2 (22%)	0	2	1 (14%)	1 (14%)	NR	NR
Kumar et al. (2016) [3]	14	0	0	0	0	0	0	0
Rajan et al. (2016) [4]	33	14 (42.4%)^†^	—^†^	—^†^	3	NR	NR	NR
Yadav et al. (2023) [12]	27	5 (18.5%)	4	1	12 (44.4%)	8/19 LB	NR	NR
Jamil et al. (2024) [13]	9	1	0	1	1	NR	NR	NR
Thomas et al. (2024) [14]	9	1	NR	NR	NR	NR	NR	NR
Rajasab and Zaki (2025) [15]	34	12 (35.3%)^†^	—^†^	—^†^	6 (17.6%)	NR	NR	NR
Zhao et al. (2025) [7]	260	57 (21.9%)	21	36	33 (12.7%)	NR	NR	NR
Meena et al. (2016) [16]	6	3	1	2	2	2	NR	1
Sheokand et al. (2021) [17]	3	1	1	0	1	1	NR	NR
Arora (2024) [18]	8	2	1	1	1	NR	NR	NR
Bahadur et al. (2024) [19]	5	1	1	0	2	1	NR	2
Selvan et al. (2024) [20]	2	0	0	0	2	2	0	2
Watthanaworawit et al. (2016) [21]	1	1	1	0	0	NR	NR	NR
Verma et al. (2017) [6]	1	1	1	0	0	NR	NR	NR
Gupta et al. (2021) [22]	1	1	1	0	0	NR	NR	NR
Rengaraj et al. (2021) [23]	1	0	0	0	1	1	1	1
Singh et al. (2021) [24]	1	0	0	0	1	1	0	NR
Tshering et al. (2021) [25]	1	0	0	0	1	1	0	NR
Singla et al. (2023) [26]	1	1	1	0	0	NR	NR	NR
Qin et al. (2024) [27]	1	1	0	1	0	NR	NR	NR
Nazreen et al. (2025) [28]	1	0	0	0	0	0	0	0

Preterm birth was reported in several studies, with frequencies ranging from 0% [3] to 44.4% (12/27) [12]. Other reported estimates included 17.6% [15] and 12.7% [7]. Low birth weight was documented in 8 of 19 live-born infants in one cohort [12] and in both neonates reported in a two-case series of pregnancies complicated by fetal growth restriction [20]. Neonatal death was uncommon but was reported in isolated cases, including one early neonatal death following delivery of an extremely low birth weight, growth-restricted infant [23]. NICU admission was reported in a small number of studies and was most commonly associated with prematurity or low birth weight [16,19,20].

Treatment

Azithromycin was the most frequently reported treatment for scrub typhus during pregnancy, typically administered at 500 mg once daily for five to seven days [4,12,15,18]. In the largest multicenter cohort, 214 of 260 (82.3%) women received a macrolide antibiotic. The authors reported that macrolide use was associated with a lower risk of adverse fetal outcomes and observed a decline in the stillbirth rate from 12.8% during 2014-2018 to 3.1% during 2019-2023, concurrent with increased macrolide use (p = 0.01) [7].

Doxycycline was infrequently used during pregnancy and, where reported, was reserved for the postpartum period or avoided during antenatal care [13]. Several case reports described initial empirical treatment with broad-spectrum antibiotics for alternative suspected bacterial infections, with azithromycin introduced only after laboratory confirmation of scrub typhus [21,22].

The diagnostic methods and treatment regimens reported across the included studies are summarized in Table 5.

**Table 5:** Diagnostic methods and treatment regimens reported across included studies. BAL = broncheoalveolar lavage; CRRT = continuous renal replacement therapy; ECMO = extracorporeal membrane oxygenation; ELISA = enzyme-linked immunosorbent assay; HEV = hepatitis E virus; IFA = immunofluorescence assay; NGS = next-generation sequencing; PCR = polymerase chain reaction; RDT = rapid diagnostic test

Study	Diagnostic method(s)	Treatment regimen	Timing/Access notes
Kumar et al. (2016) [3]	IgM ELISA (InBios)	Azithromycin 500 mg daily × 5 d, from diagnosis	Uniform early treatment; no deaths
Rajan et al. (2016) [4]	Clinical + eschar and/or IgM ELISA	Intravenous azithromycin; doxycycline postpartum in 33%	Illness >7 d predicted poor outcome
Yadav et al. (2023) [12]	IgM ELISA (OD ≥0.468)	Oral azithromycin 500 mg × 7 d (antenatal)	—
Jamil et al. (2024) [13]	Weil–Felix (OXK ≥1:160) / ICT	Azithromycin; doxycycline avoided antenatally	No eschar in any case
Rajasab and Zaki (2025) [15]	WHO criteria + IgM ELISA	Azithromycin 500 mg × 7 d	Each day of delay raised the odds of a poor outcome
Zhao et al. (2025) [7]	Weil–Felix, IFA, PCR, isolation	Macrolides in 214 of 260 (82.3%); chloramphenicol in some	Macrolide use is associated with better fetal outcomes
Meena et al. (2016) [16]	IgM ELISA (WHO)	Azithromycin 500 mg × 7 d (antenatal)	—
Arora (2024) [18]	Serum IgM ELISA	Azithromycin 500 mg daily × 5 d	5 uneventful deliveries
Bahadur et al. (2024) [19]	RDT (SD Bioline)	Azithromycin 500 mg twice daily × 5 d	Delayed access (hilly terrain)
Selvan et al. (2024) [20]	IgM ELISA	Azithromycin 500 mg × 5 d + antenatal corticosteroids	—
Watthanaworawit et al. (2016) [21]	RDT + convalescent serology	Azithromycin (initially omitted, then added)	Delay linked to deterioration
Verma et al. (2017) [6]	IgM ELISA + nested PCR	Supportive; acute-liver-failure care	Fatal; HEV co-infection
Gupta et al. (2021) [22]	IgM ELISA + eschar	Empirical broad-spectrum → azithromycin	Delay; fetal demise at presentation
Rengaraj et al. (2021) [23]	Real-time PCR	Doxycycline postpartum (from day 3)	Diagnosed postpartum
Singh et al. (2021) [24]	IgM ELISA	Azithromycin 500 mg daily × 5 d	Initially misdiagnosed
Singla et al. (2023) [26]	ELISA (fever panel)	Azithromycin; intensive organ support	Eschar missed initially; near-miss
Qin et al. (2024) [27]	NGS of BAL + blood	Doxycycline + azithromycin; ECMO, CRRT	NGS enabled diagnosis
Nazreen et al. (2025) [28]	Postoperative fever-panel PCR	Doxycycline; supportive	Fatal; diagnosed post-cesarean

Predictors of Poor Outcome

Only two studies formally evaluated predictors of adverse maternal and fetal outcomes using regression analyses. In a South Indian cohort, illness duration exceeding seven days before initiation of appropriate treatment was associated with an increased likelihood of poor outcomes (odds ratio (OR) = 2.46; 95% confidence interval (CI) = 1.6-85.9; p = 0.01) [4]. In a cohort from Telangana, each additional day of illness before treatment was independently associated with adverse outcomes (adjusted OR = 1.48, 95% CI = 1.05-2.09; p = 0.025), while lower serum albumin was also identified as an independent predictor [15].

The largest multicenter cohort similarly reported that earlier gestational age at infection and delayed initiation of macrolide therapy were associated with poorer fetal outcomes [7]. A summary of the reported predictors of adverse outcomes is presented in Table 6.

**Table 6:** Reported predictors of adverse maternal and fetal outcomes. CI = confidence interval; OR = odds ratio

Study	Predictor	Effect estimate	Outcome
Rajan et al. (2016) [4]	Illness duration >7 days	OR = 2.46 (95% CI: 1.6–85.9); p = 0.01	Poor maternal/fetal outcome
Rajasab and Zaki (2025) [15]	Illness duration (per day)	Adjusted OR = 1.48 per day (95% CI: 1.05–2.09); p = 0.025	Poor outcome; independent predictor
Rajasab and Zaki (2025) [15]	Lower serum albumin	Independent predictor (study model)	Poor outcome
Zhao et al. (2025) [7]	Earlier gestational age at infection	Inverse correlation with adverse fetal outcome	Higher fetal risk with earlier infection
Zhao et al. (2025) [7]	Macrolide therapy	Protective association	Reduced poor fetal outcome

Risk-of-Bias (Quality) Assessment

Detailed quality appraisal results are provided in Supplementary Material S2. Of the nine observational studies, three were assessed as having a low risk of bias [7,11,14], four as having a moderate risk of bias [3,4,12,15], and two as having a high risk of bias; the retrospective mortality audit by Halder et al., which lacked a defined cohort denominator and did not ascertain non-fatal maternal or fetal outcomes [10], and the study by Jamil et al., in which five of eleven items were rated No or Unclear, including an unclear case definition, lack of adjustment for confounding, and incomplete follow-up [13]. All nine case reports fulfilled most of the JBI quality assessment criteria and were judged to be low risk of bias [6,21-28]. Among the five case series, two were rated as high quality [18,19] and three as moderate quality [16,17,20], with the most common limitations being unclear consecutive recruitment and incomplete follow-up. Overall, most included studies were judged to be of low-to-moderate risk of bias. However, the evidence base was limited by the predominance of observational studies, case series, and case reports, most of which were conducted at single centers with relatively small sample sizes.

Discussion

This systematic review synthesized evidence from 23 studies involving 434 pregnant women with laboratory-confirmed scrub typhus. Overall, scrub typhus during pregnancy was associated with substantial maternal and fetal morbidity, including critical maternal illness, maternal death, fetal loss, and preterm birth. However, the frequency of these outcomes varied considerably across studies, reflecting differences in study populations, healthcare settings, diagnostic approaches, disease severity, and treatment practices. Despite the inclusion of one large multicenter cohort, the available evidence remains dominated by small, facility-based observational studies and case reports, predominantly from India, leaving the true community-level burden uncertain. A similar systematic review by Sengupta et al. [29] examined fetal outcomes among pregnant women with leptospirosis and rickettsial infections and identified 22 scrub typhus-specific studies contributing 176 cases, with fetal loss in 53 (30.1%) and neonatal death in three. The present review extends this work by focusing exclusively on scrub typhus, evaluating maternal as well as fetal outcomes in greater depth, including maternal mortality, critical illness, and prognostic factors for adverse outcomes, and incorporating an updated search through December 2025.

Maternal mortality varied markedly across studies. All reported maternal deaths occurred in Indian studies, whereas no maternal deaths were observed in the largest multicenter cohort from China [7]. This difference is more likely to reflect variation in case mix, referral patterns, and timing of presentation than geographic differences in pathogen virulence. Most Indian studies were conducted in tertiary referral centers, where women often presented with advanced disease, and several authors identified delayed diagnosis and initiation of appropriate therapy as important contributors to adverse outcomes [6,22,28]. Consistent with these observations, the two studies that formally evaluated prognostic factors identified delayed treatment as an independent predictor of adverse maternal and fetal outcomes [4,15], while the largest cohort reported poorer fetal outcomes among women receiving delayed macrolide therapy [7]. Collectively, these findings suggest that timely diagnosis and treatment are likely to be key determinants of clinical outcome.

Fetal complications were reported frequently, with fetal loss and preterm birth representing the most common adverse outcomes. In the largest cohort, fetal loss occurred more commonly among women infected earlier in pregnancy, suggesting greater fetal vulnerability during early gestation [7]. Although none of the included studies demonstrated confirmed vertical transmission of *O. tsutsugamushi*, maternal hyperpyrexia, systemic vasculitis, placental hypoperfusion, hypoxemia, and coagulation abnormalities provide biologically plausible mechanisms through which maternal infection may adversely affect pregnancy.

Delayed recognition emerged as a recurring challenge across the included studies. Fever was the predominant presenting symptom, whereas the characteristic eschar was reported inconsistently and was frequently absent. Clinical manifestations often overlapped with other infectious and obstetric conditions, including pneumonia, COVID-19, acute pyelonephritis, HELLP syndrome, and puerperal sepsis [17,22-25]. In endemic settings, where scrub typhus coexists with malaria, dengue, leptospirosis, and enteric fever, this diagnostic overlap may delay initiation of appropriate therapy.

The treatment findings further support the importance of early recognition. Azithromycin was the most frequently used antimicrobial across the included studies, and observational evidence drawn mainly from two small single-center regression analyses [4,15] and an uncontrolled before/after comparison in the largest cohort [7] consistently suggested more favorable maternal and fetal outcomes with earlier treatment. Given the small number of studies, their single-center design, and the absence of controlled comparisons, these findings should be regarded as hypothesis-generating rather than as evidence supporting a specific treatment recommendation, and warrant confirmation in prospective, multicenter studies before informing empirical treatment practice.

Strengths and Limitations

This review has several strengths. It followed a prospectively registered protocol and adhered to the PRISMA 2020 and SWiM reporting guidelines. A comprehensive search strategy was implemented across five bibliographic databases, four clinical trial registries, and two additional sources (Google Scholar, as a source of grey literature, and the ONOS portal). Independent selection of studies, data extraction, and methodological quality assessment minimized the risk of reviewer bias. A conservative data extraction strategy was adopted, in which only explicitly reported outcomes were extracted, thereby reducing the potential for misclassification.

Several limitations should be considered. All included studies were facility-based, predominantly from tertiary referral centers, and therefore are likely to overrepresent severe disease while underestimating milder community-managed infections. The available evidence was heavily concentrated in India. The search was also restricted to English-language publications; relevant non-English literature, particularly from China, may therefore have been missed, limiting the generalizability of the findings. Considerable heterogeneity in study design, diagnostic methods, treatment protocols, and outcome definitions precluded quantitative synthesis. Furthermore, several studies relied on Weil-Felix testing or single IgM ELISA assays without confirmatory molecular testing, introducing the possibility of diagnostic misclassification. Finally, the predominance of case reports and small case series may have resulted in overrepresentation of severe clinical presentations.

Implications for Practice

The findings of this review have important implications for clinical practice in endemic settings. Scrub typhus should be considered in the differential diagnosis of acute undifferentiated fever during pregnancy, particularly when accompanied by thrombocytopenia or abnormal liver function tests. Careful examination for an eschar remains important despite its inconsistent presence. Given the association between delayed treatment and adverse outcomes, empirical azithromycin should be considered when clinical suspicion is high, and laboratory confirmation is likely to be delayed. Women with severe disease require multidisciplinary management, including critical care support where indicated and close fetal surveillance throughout the course of illness.

Future Research

Future research should prioritize prospective, multicenter, population-based studies using standardized laboratory confirmation and harmonized outcome definitions to better estimate the burden of scrub typhus during pregnancy across endemic regions. Greater geographical representation beyond India is required to improve external validity. Standardized core outcome sets would facilitate future quantitative synthesis, while placental studies, cord blood PCR, neonatal serology, and placental histopathology may help clarify whether vertical transmission occurs. Improved point-of-care diagnostic tests also have the potential to reduce diagnostic delays and improve maternal and fetal outcomes.

## Conclusions

Scrub typhus during pregnancy is associated with a substantial, though variable, burden of maternal and fetal morbidity. The available evidence, derived exclusively from healthcare facility-based studies, suggests that clinical outcomes are influenced more by the timeliness of diagnosis and initiation of appropriate therapy than by geographic location, although the true community-level burden remains unknown. In endemic settings, clinicians should maintain a high index of suspicion and consider empirical azithromycin therapy when scrub typhus is strongly suspected, particularly when laboratory confirmation is unavailable or likely to be delayed. Well-designed prospective, multicenter, population-based studies using standardized diagnostic criteria and outcome definitions are needed to better define the epidemiology of scrub typhus in pregnancy and inform evidence-based management strategies.

## Appendices

Appendix 1: Search strategies

Results were limited to English-language, full-text articles published between January 1, 2015, and December 31, 2025. The core concepts were the pathogen (*Orientia tsutsugamushi*/scrub typhus) combined with pregnancy terms and with outcome or prevalence terms. Five bibliographic databases and four trial registries were searched, and two additional sources were searched separately: Google Scholar, for grey literature, and the ONOS portal.

**Table 7:** Supplementary Table S1: Search strategies.

Source	Search string	Filters/limits	Records retrieved
1. PubMed	((“Scrub Typhus”[Mesh] OR “scrub typhus”[tiab] OR “Orientia tsutsugamushi”[tiab] OR Orientia[tiab] OR rickettsiosis[tiab] OR rickettsial[tiab]) AND (“Pregnancy”[Mesh] OR pregnan*[tiab] OR pregnant[tiab] OR matern*[tiab] OR gestation*[tiab] OR antenatal[tiab] OR prenatal[tiab] OR obstetric*[tiab] OR gravid*[tiab] OR parturient*[tiab] OR perinatal[tiab] OR fetal[tiab] OR foetal[tiab] OR neonatal[tiab]) AND (prevalence[tiab] OR incidence[tiab] OR epidemiology[tiab] OR outcome*[tiab] OR complication*[tiab] OR adverse[tiab] OR maternal[tiab] OR fetal[tiab] OR foetal[tiab] OR neonatal[tiab] OR perinatal[tiab] OR morbidity[tiab] OR mortality[tiab] OR miscarriage[tiab] OR abortion[tiab] OR stillbirth[tiab] OR “fetal death”[tiab] OR IUFD[tiab] OR preterm[tiab] OR prematurity[tiab] OR “preterm birth”[tiab] OR “low birth weight”[tiab] OR LBW[tiab] OR "small for gestational age"[tiab] OR SGA[tiab] OR NICU[tiab] OR sepsis[tiab] OR thrombocytopenia[tiab] OR hepatitis[tiab] OR ARDS[tiab] OR AKI[tiab])) Basic-search equivalent: scrub typhus AND pregnancy	English 2015/01/01–2025/12/31	70
2. Ovid Embase	(‘scrub typhus’/exp OR ‘orientia tsutsugamushi’) AND (‘pregnancy’/exp OR ‘pregnant woman’/exp OR antenatal OR maternal) AND (prevalence OR incidence OR outcome OR fetal outcome OR maternal complication OR mortality) Basic-search equivalent: scrub typhus AND pregnancy.	English yr = “2015–2025”	31
3. CINAHL (via EBSCOhost)	(MH “Scrub Typhus” OR TX “scrub typhus” OR TX “Orientia tsutsugamushi” OR TX tsutsugamushi) AND (MH “Pregnancy+” OR MH “Pregnant Women” OR TX pregnan* OR TX maternal OR TX obstetric* OR TX antenatal OR TX prenatal OR TX gestation* OR TX gravid*) AND (MH “Pregnancy Outcome+” OR MH “Pregnancy Complications+” OR TX prevalence OR TX incidence OR TX outcome* OR TX complication* OR TX “maternal outcome*” OR TX “fetal outcome*” OR TX “foetal outcome*” OR TX “pregnancy outcome*” OR TX “birth outcome*” OR TX morbidity OR TX mortality) Basic-search equivalent: scrub typhus AND pregnancy	English 1 Jan 2015–31 Dec 2025	78
4. ONOS portal (One Nation One Subscription)	(“scrub typhus” OR “Orientia tsutsugamushi”) AND (pregnan* OR pregnancy OR pregnant OR maternal OR gestation OR antenatal OR prenatal OR obstetric*) Publisher-access gateway with a unified search box; does not support field-tagged syntax. Simplified string used and results screened manually	—	20
5. MEDLINE Ultimate (via EBSCOhost)	(MH “Scrub Typhus+” OR TX “scrub typhus” OR TX “Orientia tsutsugamushi” OR TX Orientia OR TX rickettsial) AND (MH “Pregnancy+” OR TX pregnan* OR TX pregnant OR TX matern* OR TX gestation* OR TX antenatal OR TX prenatal OR TX obstetric* OR TX gravid* OR TX parturient* OR TX perinatal OR TX fetal OR TX foetal OR TX neonatal) AND (MH “Pregnancy Outcome+” OR MH “Pregnancy Complications+” OR TX prevalence OR TX incidence OR TX epidemiology OR TX outcome* OR TX complication* OR TX adverse OR TX maternal OR TX fetal OR TX foetal OR TX neonatal OR TX perinatal OR TX morbidity OR TX mortality OR TX miscarriage OR TX abortion OR TX stillbirth OR TX “fetal death” OR TX IUFD OR TX preterm OR TX prematurity OR TX “preterm birth” OR TX “low birth weight” OR TX LBW OR TX “small for gestational age” OR TX SGA OR TX NICU OR TX sepsis OR TX thrombocytopenia OR TX hepatitis OR TX ARDS OR TX AKI) Basic-search equivalent: scrub typhus AND pregnancy	English 1 Jan 2015–31 Dec 2025	217
6. ClinicalKey	Search term: “scrub typhus in pregnancy” Searched across All Content (including Full Text and MEDLINE)	English 1 Jan 2015–31 Dec 2025	627
7. Trial registries	Terms: “scrub typhus” AND “pregnancy” Searched: WHO ICTRP, ClinicalTrials.gov, EU Clinical Trials Register, ISRCTN. ISRCTN returned 3; the other three returned none	—	3
8. Google Scholar	(“scrub typhus” OR “Orientia tsutsugamushi”) AND (pregnancy OR pregnant) AND (maternal outcomes OR fetal outcomes OR complications OR prevalence) Does not support nested Boolean operators or field tags; used for supplementary retrieval and reference chaining	Custom range 2015–2025	719
Total records retrieved	-	-	1,765

Appendix 2: Risk-of-bias (JBI) appraisal

Each study was appraised with the JBI checklist matched to its design. Items are coded Y (yes), N (no), and U (unclear). Overall judgements were reached by consensus and correspond exactly to the Results section in the main manuscript. Studies are listed in the citation order used in the manuscript.

**Table 8:** Supplementary Table S2.1: JBI appraisal: cohort and observational studies (11 items).

Study	1	2	3	4	5	6	7	8	9	10	11	Risk of bias
Halder et al. (2015) [10]	Y	Y	Y	U	U	N	Y	Y	N	N	Y	High
Chansamouth et al. (2016) [11]	Y	Y	Y	Y	Y	Y	Y	Y	Y	U	Y	Low
Kumar et al. (2016) [3]	Y	Y	Y	Y	U	Y	Y	Y	Y	U	Y	Moderate
Rajan et al. (2016) [4]	Y	Y	Y	Y	Y	Y	Y	Y	N	N	Y	Moderate
Yadav et al. (2023) [12]	Y	Y	Y	Y	N	Y	Y	Y	U	U	Y	Moderate
Jamil et al. (2024) [13]	N	Y	Y	N	N	Y	Y	Y	N	N	Y	High
Thomas et al. (2024) [14]	Y	Y	Y	Y	Y	Y	Y	Y	Y	N	Y	Low
Rajasab and Zaki (2025) [15]	Y	Y	Y	Y	Y	Y	Y	Y	U	U	Y	Moderate
Zhao et al. (2025) [7]	Y	Y	Y	Y	Y	Y	Y	Y	Y	U	Y	Low

**Table 9:** Supplementary Table S2.2: JBI appraisal: case series (10 items).

Study	1	2	3	4	5	6	7	8	9	10	Overall
Meena et al. (2016) [16]	Y	Y	Y	Y	Y	Y	Y	U	U	Y	Moderate
Sheokand et al. (2021) [17]	Y	Y	Y	Y	Y	Y	Y	N	U	Y	Moderate
Arora (2024) [18]	Y	Y	Y	Y	Y	Y	Y	Y	U	Y	Low
Bahadur et al. (2024) [19]	Y	Y	Y	Y	Y	Y	Y	Y	U	Y	Low
Selvan et al. (2024) [20]	Y	Y	Y	Y	Y	Y	Y	U	U	Y	Moderate

**Table 10:** Supplementary Table S2.3. JBI appraisal: case reports (8 items).

Study	1	2	3	4	5	6	7	8	Overall
Watthanaworawit et al. (2016) [21]	Y	Y	Y	Y	Y	Y	Y	Y	Low
Verma et al. (2017) [6]	Y	Y	Y	Y	Y	Y	Y	Y	Low
Gupta et al. (2021) [22]	Y	Y	Y	Y	Y	Y	Y	Y	Low
Rengaraj et al. (2021) [23]	Y	Y	Y	Y	Y	Y	Y	Y	Low
Singh et al. (2021) [24]	Y	Y	Y	Y	Y	Y	Y	Y	Low
Tshering et al. (2021) [25]	Y	Y	Y	Y	Y	Y	Y	Y	Low
Singla et al. (2023) [26]	Y	Y	Y	Y	Y	Y	Y	Y	Low
Qin et al. (2024) [27]	Y	Y	Y	Y	Y	Y	Y	Y	Low
Nazreen et al. (2025) [28]	Y	Y	Y	Y	Y	N	Y	Y	Low

 Appendix 3: Data extraction sheet

**Table 11:** Supplementary Material S3: Complete data extraction dataset. AKI = acute kidney injury; ARDS = acute respiratory distress syndrome; CRRT = continuous renal replacement therapy; DIC = disseminated intravascular coagulation; ECMO = extracorporeal membrane oxygenation; HEV = hepatitis E virus; ICU = intensive care unit; LFT = liver function test; MODS = multiorgan dysfunction syndrome; NR = not reported

Study	N	Death	ICU	ARDS	MODS	AKI	DIC	Hepatic/other	Fetal loss	IUD/SB	Abortion	Preterm	LBW	Neonatal death	NICU
Halder et al. (2015) [10]	6*	6	NR	NR	NR	NR	NR	Audit-level only	NR	NR	NR	NR	NR	NR	NR
Chansamouth et al. (2016) [11]	9	0	NR	NR	NR	NR	NR	Not disaggregated for ST	2	0	2	1	1	NR	NR
Kumar et al. (2016) [3]	14	0	NR	4	4	5	NR	Hepatic dysfunction 11; sepsis 10	0	0	0	0	0	0	0
Rajan et al. (2016) [4]	33	1	23	NR	5	NR	NR	Hepatosplenomegaly 4	14	comp	comp	3	NR	NR	NR
Yadav et al. (2023) [12]	27	1	8	1	4	2	NR	Hyperbilirubinemia 13	5	4	1	12	8/19 LB	NR	NR
Jamil et al. (2024) [13]	9	0	NR	NR	2	NR	NR	Hepatitis 1; eclampsia 1	1	0	1	1	NR	NR	NR
Thomas et al. (2024) [14]	9	0	2	NR	NR	NR	NR	Not broken out for ST	1	NR	NR	NR	NR	NR	NR
Rajasab and Zaki (2025) [15]	34	0	15	2	NR	3	NR	Jaundice 5; pneumonia 4	12	comp	comp	6	NR	NR	NR
Zhao et al. (2025) [7]	260	0	8	NR	NR	35	2	Hepatic dysfunction 105; pneumonia 33	57	21	36	33	NR	NR	NR
Meena et al. (2016) [16]	6	1	≥2	2	2	2	1	Hepatitis 4	3	1	2	2	2	NR	1
Sheokand et al. (2021) [17]	3	0	1	1	1	1	1	Deranged LFTs	1	1	0	1	1	NR	NR
Arora (2024) [18]	8	0	3	2	1	2	NR	Not separately reported	2	1	1	1	NR	NR	NR
Bahadur et al. (2024) [19]	5	1	1	1	1	0	NR	Deranged LFTs in all 5	1	1	0	2	1	NR	2
Selvan et al. (2024) [20]	2	0	0	1	1	1	NR	Hepatitis 1	0	0	0	2	2	0	2
Watthanaworawit et al. (2016) [21]	1	0	1	1	1	NR	1	Septic shock	1	1	0	0	NR	NR	NR
Verma et al. (2017) [6]	1	1	1	NR	1	NR	1	Acute liver failure (HEV)	1	1	0	0	NR	NR	NR
Gupta et al. (2021) [22]	1	0	1	1	1	1	0	Hypoxemic respiratory failure	1	1	0	0	NR	NR	NR
Rengaraj et al. (2021) [23]	1	0	NR	0	NR	1	NR	Hepatitis; HELLP mimic	0	0	0	1	1	1	1
Singh et al. (2021) [24]	1	0	NR	1	1	1	NR	Pneumonia; hepatomegaly	0	0	0	1	1	0	NR
Tshering 2021 [25]	1	0	NR	1	NR	NR	NR	Permanent hearing loss	0	0	0	1	1	0	NR
Singla et al. (2023) [26]	1	0	1	1	1	NR	NR	Near-miss; thrombocytopenia	1	1	0	0	NR	NR	NR
Qin et al. (2024) [27]	1	0	1	1	1	NR	NR	ECMO + CRRT	1	0	1	0	NR	NR	NR
Nazreen et al. (2025) [28]	1	1	1	1	1	1	NR	Ogilvie syndrome	0	0	0	0	0	0	0

 Appendix 4: Outcome definitions

**Table 12:** Supplementary Material S4: Outcome definitions. AKI = acute kidney injury; ARDS = acute respiratory distress syndrome; DIC = disseminated intravascular coagulation; LBW = low birth weight; MODS = multiorgan dysfunction syndrome; NICU = neonatal intensive care unit

Outcome	Operational definition used in this review
Maternal death	Death of the mother during pregnancy, delivery, or the puerperium, attributed by the source study to scrub typhus
ICU admission	Documented admission to an intensive care or high-dependency unit. Ventilatory support not explicitly described as ICU care was recorded as not reported
ARDS/Respiratory distress	Acute respiratory distress syndrome, or respiratory distress explicitly labelled as such. Breathlessness not described against ARDS criteria was recorded as not reported
Hepatitis/Hepatic dysfunction	Hepatitis or hepatic dysfunction was explicitly reported. Isolated transaminase or bilirubin elevation, not labelled as hepatitis, was noted separately and not counted as this outcome
MODS	MODS, as defined and labelled by the source study
AKI	AKI or renal dysfunction, as defined by the source study
Coagulopathy/DIC	DIC, or coagulopathy, is explicitly labelled as such. Isolated thrombocytopenia was not counted as DIC
Total fetal loss	Any pregnancy loss, intrauterine death, stillbirth, or spontaneous abortion, combined where the source did not separate them
Intrauterine death/Stillbirth	Fetal death in utero at or beyond the locally applied threshold of viability, as reported
Spontaneous abortion	Pregnancy loss before the locally applied threshold of viability, as reported
Preterm birth	Live birth before 37 completed weeks of gestation
LBW	Birth weight below 2,500 g; the denominator was taken as live births where specified
Neonatal death	Death of a live-born infant within the neonatal period, as reported
NICU admission	Documented admission of the neonate to a NICU
Prevalence	Proportion of screened or admitted febrile pregnant women with confirmed scrub typhus, using the denominator reported by the source study

 Appendix 5: PRISMA 2020 checklist

**Table 13:** Supplementary Material S5: PRISMA 2020 checklist.

Item	PRISMA 2020 checklist item	Location in manuscript
1	Title identifies the report as a systematic review	Title
2	Structured abstract	Abstract
3	Rationale	Introduction §1
4	Objectives	Introduction §1 (Objectives)
5	Eligibility criteria	Methodology
6	Information sources	Methodology; Supplement S1
7	Search strategy	Methodology; Supplement S1
8	Selection process	Methodology; Figure 1
9	Data-collection process	Methodology; Supplement S4
10	Data items	Methodology; Supplements S4 and S5
11	Risk-of-bias assessment	Methodology; Supplement S2
12	Effect measures	Methodology (proportions and ranges; no pooling)
13	Synthesis methods	Methodology (SWiM)
14	Reporting-bias assessment	Methodology; Discussion
15	Certainty assessment	Methodology (GRADE not applied — outcomes not pooled)
16	Study selection results	Results; Figure 1
17	Study characteristics	Results; Table 1
18	Risk of bias in studies	Results; Supplement S2
19	Results of individual studies	Tables 1–4; Supplement S4
20	Results of syntheses	Results; Tables 2–6
21	Reporting biases	Discussion
22	Certainty of evidence	Discussion
23	Discussion	Discussion; Conclusion
24	Registration and protocol	Title page; Methodology
25	Support/Funding	Declarations
26	Competing interests	Declarations
27	Availability of data, code and other materials	Declarations; Supplement S4

Appendix 6: SWiM checklist 

**Table 14:** Supplementary Material S6: SWiM checklist.

Item	SWiM item	How addressed
1	Grouping studies for synthesis	Grouped by design (cohort or observational, case series, case reports) and, within the results, by outcome domain - Methodology; Results
2	Standardized metric	Counts and proportions (n/N) with study-level percentages and ranges; no transformation to a common metric - Methodology; Tables 3 and 4
3	Synthesis method	Structured narrative synthesis; no meta-analysis, with reasons given - Methodology
4	Criteria for prioritizing results	Larger cohorts at lower risk of bias were given the greatest interpretive weight - Results; Discussion
5	Investigation of heterogeneity	Explored qualitatively by design, setting, diagnostic method and timeliness of treatment - Results; Discussion
6	Certainty/Limitations of the synthesis	Addressed narratively; no formal GRADE rating, as outcomes were not pooled - Methodology; Discussion
7	Data presentation methods	Study-level tables (Tables 1–6) and the complete extraction dataset (Supplement S4)
8	Reporting biases	Selection and publication bias toward severe cases discussed - Discussion
9	Description of the effect	Direction and range of outcomes reported without a single pooled estimate - Results
